# Cardioprotective Vaccination Effects: Association Between Vaccination Knowledge, Risk Perception, and Vaccination Intention in Older Patients with Pre-Existing Conditions

**DOI:** 10.3390/vaccines14100901

**Published:** 2026-10-14

**Authors:** Sebastian Born, Carl Zimmermann, Norman Rose, Mathias W. Pletz, Konstantin G. Heimrich, Carolin Fleischmann-Struzek

**Affiliations:** 1 Institute of Infectious Diseases and Infection Control, Jena University Hospital, Friedrich Schiller University Jena, 07743 Jena, Germany; 2 Center for Sepsis Control and Care, Jena University Hospital, Friedrich Schiller University Jena, 07743 Jena, Germany; 3 Department of Geriatrics, Jena University Hospital, Friedrich Schiller University Jena, 07743 Jena, Germany

**Keywords:** awareness, cardioprotective effect, cardiovascular disease, influenza, patient education, pneumococcal disease, risk perception, vaccination

## Abstract

Background/Objectives: Cardiovascular diseases (CVDs) remain a major contributor to morbidity, mortality, and healthcare costs worldwide. Acute respiratory infections such as influenza and pneumococcal disease can precipitate severe cardiovascular events, while vaccination against these pathogens offers both infection-related and cardioprotective benefits. The extent to which individuals are aware of these associations and how such knowledge shapes risk perception and vaccination intention remains unclear. Methods: In a cross-sectional survey conducted between July and September 2025 in Germany, 101 geriatric patients and individuals with pre-existing cardiovascular conditions were assessed regarding their knowledge of infection-related cardiovascular risks and cardioprotective vaccination benefits, risk perception, and vaccination intention. Results: While 64.4% of participants recognized the increased cardiovascular risk associated with acute respiratory infections, only 40.6% were aware of the cardioprotective benefits of vaccination. Knowledge of infection-related cardiovascular risk was linked to higher perceived disease severity, whereas awareness of cardioprotective vaccination effects was associated with greater perceived usefulness of influenza and pneumococcal vaccinations. Crucially, only knowledge of cardioprotective benefits (*b*_y_(flu) = 0.46, *b*_y_(pneu) = 0.45), but not knowledge of infection-related cardiovascular risk (*b*_y_(flu) = 0.08, *b*_y_(pneu) = 0.34) was significantly associated with higher vaccination intention. Conclusions: These findings highlight substantial knowledge gaps regarding the cardioprotective benefits of influenza and pneumococcal vaccination. These findings support further evaluation of whether communication emphasizing potential cardioprotective benefits can influence vaccination intentions and subsequent vaccine uptake in prospective or interventional studies:

## 1. Introduction

Cardiovascular diseases are among the most common causes of morbidity and mortality worldwide [1]. In Germany, cardiovascular diseases (CVDs) represent the leading cause of death, with approximately 340,000 deaths per year [2]. Despite declining mortality rates, the burden of disease for patients, relatives, and the healthcare system remains substantial [3]. In 2017, CVDs were major contributors to the national disease burden in Germany, accounting for 2321 disability-adjusted life years (DALYs) per 100,000 inhabitants [4]. Moreover, they caused the highest direct healthcare costs of all diagnostic groups in the German healthcare system, amounting to 64.6 billion euros in 2023 [5].

Previous studies suggest that pathogens causing acute respiratory infections, such as influenza and pneumococci, can significantly increase the risk of cardiovascular complications such as myocardial infarction, sudden cardiac death, or stroke [6,7,8,9]. Conversely, randomized controlled trials and recent meta-analyses found that influenza and pneumococcal vaccination not only protect the general population and high-risk patients against influenza and pneumonia, but also reduce the risk of severe cardiovascular events [10,11,12,13,14]. The adjusted relative risk for cardiovascular disease was 26% lower in individuals who received an influenza vaccination compared with those who were unvaccinated [11]. The effects are even stronger in high-risk patients. In a study involving individuals with pre-existing cardiovascular disease, the rates of death due to cardiovascular causes were 2.7% versus 4.5% (hazard ratio: *HR* = 0.59; 95% CI: 0.39–0.90) in the vaccine versus placebo group [10]. For pneumococcal vaccination, a recent meta-analysis found a decreased risk for any cardiovascular event (risk ratio: *RR* = 0.91; 95% CI: 0.84–0.99) and for myocardial infarction (*RR*: 0.88; 95% CI: 0.79–0.98), with a significant protective effect observed only in individuals aged ≥65 years [14].

Influenza and pneumococcal vaccination coverage in Germany remains low, despite recommendations by the German Standing Committee on Vaccination (STIKO) for defined target populations (persons aged ≥60 years and patients with chronic conditions, including chronic cardiovascular diseases) [15] as well as reimbursement through statutory health insurance, which covers approximately 88% of the German population, and most private health insurance plans, covering together an additional 10.5%. Public health measures to promote vaccination include national vaccination recommendations, information campaigns by the Federal Institute for Public Health (BIÖG) [16], vaccination services provided through primary care and pharmacies for influenza vaccination, and incentives offered by health insurance funds for preventive measures such as vaccinations. However, in 2023, only 38% and 20% of individuals aged ≥60 years and 31% and 23% of individuals with relevant underlying medical conditions were vaccinated against influenza and pneumococci, respectively [17]. These vaccination rates fell short of the WHO target of 75% influenza vaccination coverage [18].

One possible reason for this is that the cardioprotective effects of influenza and pneumococcal vaccination have not been communicated in existing informational materials, although emphasizing these additional benefits could positively influence vaccination attitudes and increase vaccine uptake. To this end, the previous Danish interventional NUDGE-FLU studies [19,20,21] which included more than 900,000 participants, demonstrated that digitally delivered informational letters highlighting the cardioprotective benefit of influenza vaccination produced measurable improvements in vaccination uptake. Among nine formats tested, the one emphasizing the cardioprotective benefit (“cardiovascular gain frame”) was associated with the highest effectiveness [20]. However, it remains unclear whether knowledge of the cardiovascular risk of acute respiratory infections and the cardioprotective benefits of vaccination is associated with individuals’ risk perception and vaccination intention. Therefore, the study aims to address three research questions:

(1) What is the prevalence of knowledge about the increased cardiovascular risk associated with acute respiratory infections and the cardioprotective benefits of vaccination, and does this knowledge differ between individuals with and without pre-existing cardiovascular diseases?
(2) How is this knowledge related to individual risk perception?
(3) How is this knowledge associated with vaccination intention?

## 2. Methods

### 2.1. Study Design, Setting and Sample Recruitment

As part of the project “Vaccination Attitude of Risk Groups for Cardiovascular and Neurodegenerative Diseases” (VARiCaNe), a cross-sectional survey was conducted between July and September 2025 among hospitalized geriatric patients at Jena University Hospital and through self-help groups. We assessed 101 individuals with pre-existing cardiovascular conditions or aged ≥60 years with a standardized questionnaire. Persons who provided informed consent were surveyed either online or with assistance from trained student research assistants using an online questionnaire. Ethical approval for the study was obtained from the Institutional Review Board of the Friedrich Schiller University Jena (2025-3840-Bef). The reporting of the study followed the checklist for reporting of survey studies (CROSS) [22]. Because the study was observational with a resulting sample determined by the predefined recruitment period, no a priori sample-size calculation was performed.

### 2.2. Variables

We recorded age, sex, and educational level as sociodemographic variables. Educational level was categorized according to the educational degrees of Germany (low: 8–9 years of schooling; intermediate: 10 years of schooling; high: university entrance qualification or university degree). In addition, participants self-reported the presence of pre-existing cardiovascular conditions and their influenza and pneumococcal vaccination status.

#### 2.2.1. Vaccination Knowledge

We assessed vaccination knowledge with single-item measures addressing the relationship between acute respiratory infections and cardiovascular risk, as well as the cardioprotective effects of influenza and pneumococcal vaccination. These items were phrased to capture conceptual knowledge about the link between respiratory infections, vaccination, and cardiovascular risk and cardioprotective benefit. Participants had to decide whether each statement was true or false. Correct answers were coded as one, indicating that participants possessed correct knowledge of the specific concept, and incorrect answers as well as “I don’t know” responses were coded as zero.

#### 2.2.2. Risk Perception

Risk perception was conceptualized according to the Health Belief Model [23] along two dimensions: infection-related perceptions, including perceived severity of influenza and pneumococcal disease and perceived susceptibility to an influenza or pneumococci infection, and vaccination-related perceptions, comprising perceived benefits and perceived risks of the corresponding vaccination. We measured these aspects using four single-item measures from previous studies [24,25] assessing the perceived danger of the disease, perceived likelihood of contracting it, perceived benefit of vaccination, and perceived risk of vaccination. All items were rated on a five-point Likert scale ranging from 1 = very low to 5 = very high.

#### 2.2.3. Vaccination Intention

Vaccination intention represents a theoretically well-established endpoint, as it is considered an immediate predictor of actual health behavior in health psychology, among others in the Theory of Planned Behavior [26], in which intention directly predicts behavior. Vaccination intention was assessed by asking participants to indicate the likelihood of receiving the respective vaccination, specifically the likelihood of obtaining an influenza vaccination during the upcoming season and the likelihood of receiving a pneumococcal vaccination within the next six months. Responses were rated on a five-point Likert scale ranging from 1 = very low to 5 = very high.

The complete questionnaire can be found in the Supplementary Materials.

### 2.3. Analyses

Descriptive statistics were computed for sociodemographic characteristics and patient data. Means (*M*) and standard deviations (SDs) were reported for continuous variables, and frequencies and percentages for categorical variables.

To assess the prevalence of knowledge about the increased cardiovascular risk associated with acute respiratory infections and the cardioprotective benefits of vaccination, proportions were calculated. Differences in knowledge between individuals with and without pre-existing cardiovascular conditions were examined using Fisher’s exact test. To analyze how knowledge relates to individual risk perception, independent sample *t*-tests or Welch’s *t*-tests (in cases of heteroscedasticity) were applied. Knowledge about the increased cardiovascular risk served as the grouping variable for infection-related risk perception (severity, susceptibility), whereas knowledge about the cardioprotective benefits served as the grouping variable for vaccine-related perceptions (benefits, risks). As effect size measure, we calculated odds ratios (*OR*) for the Fisher’s exact test and Cohen’s *d* for the *t*-tests or Welch’s *t*-tests with 95% confidence interval (CI).

Associations between vaccination intention and knowledge were examined using linear regression models adjusted for age, sex, and educational level. Separate models were estimated for influenza and pneumococcal vaccination intention. We report y-standardized regression coefficients *b*_y_, as several predictors are dichotomous and standardizing such variables would not be meaningful.

All analyses were conducted using the statistical software R v.4.2.3 (R Foundation for Statistical Computing, Vienna, Austria). Statistical significance was set at α = 0.05. We reported unadjusted *p*-values. Missing data were handled using listwise deletion.

## 3. Results

A total of 101 respondents participated in the survey. Mean age of *M* = 71.9 years (SD = 16.7) and 22.8% had a low, 26.7% a medium, and 50.5% a high educational level. The majority of participants was female (67.0%) and had pre-existing cardiovascular conditions (61.6%). Knowledge about the increased cardiovascular risk of acute respiratory infections was present in 64.4% of participants, while 40.6% were aware of the cardioprotective benefits of vaccination. A significant difference was observed between individuals with and without cardiovascular pre-existing conditions regarding the knowledge of cardioprotective vaccination benefits (49.2% with vs. 26.3% without cardiovascular comorbidities, *OR* [95% CI] = 2.68 [1.04, 7.32], *p* = 0.035), but not regarding the increased cardiovascular risk (68.9% vs. 55.3%, *OR* [95% CI] = 1.78 [0.71, 4.49], *p* = 0.201). Vaccination rates were 63.0% for influenza and 51.1% for pneumococci. Further details can be found in Table 1.

**Figure 1:**
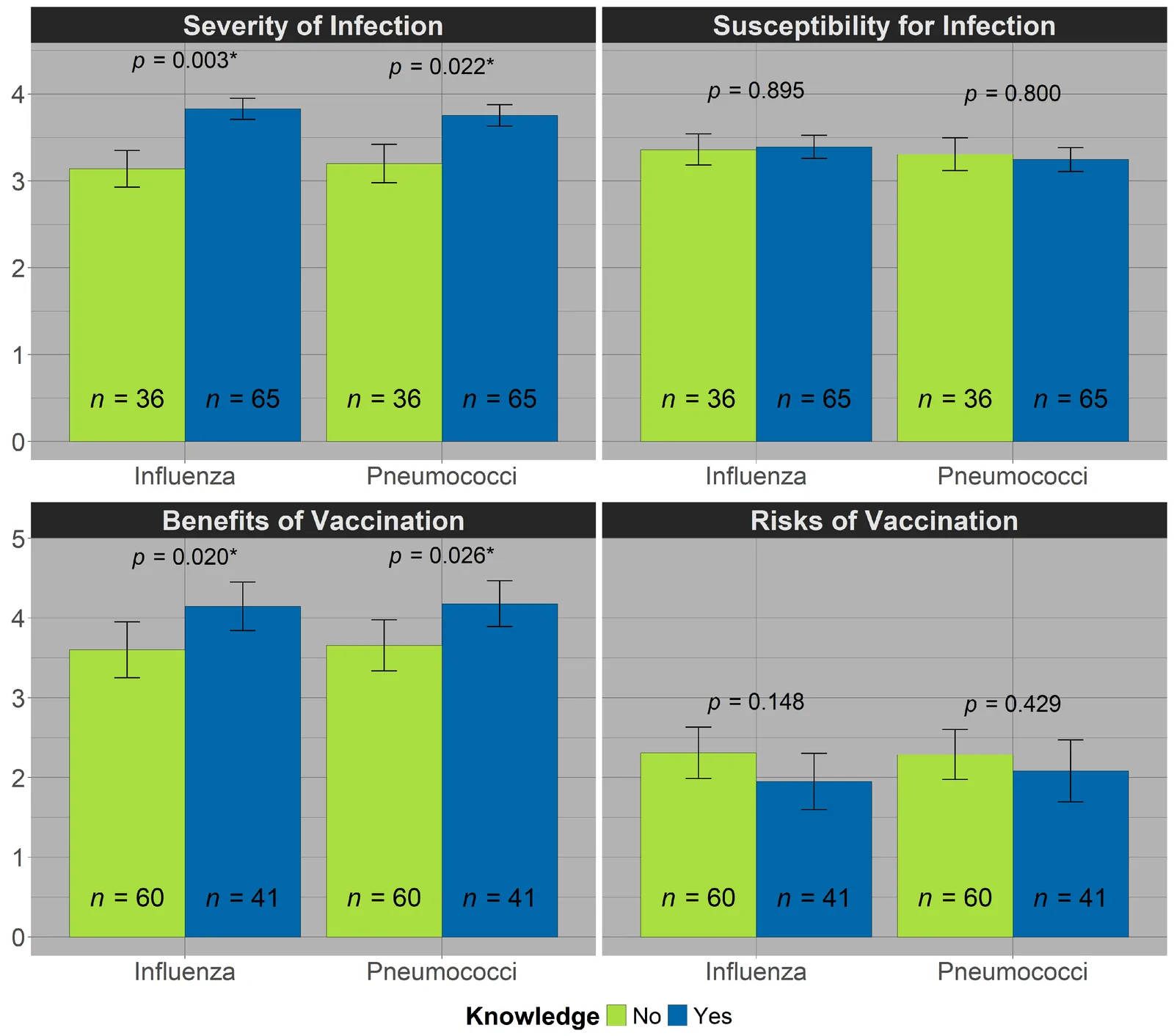
Descriptive statistics (Mean and 95% CI) and group comparisons for infection-related and vaccination-related aspects of risk perception for influenza and pneumococci depending on knowledge (*n* = 101). Note: aspects of risk perception were measured on a scale from 1 = very low to 5 = very high. Knowledge about the increased cardiovascular risk of acute respiratory infections or the cardio-protective benefit of vaccinations served as the grouping variable for infection-related and vaccination-related aspects of risk perception, respectively. Group comparisons were conducted using *t*-tests or robust Welch *t*-tests in cases of heteroscedasticity. * = significant at significance level α = 0.05.

Individuals who had knowledge about the increased cardiovascular risk of influenza and pneumococcal infections (Figure 1) rated these infections as significantly more dangerous than those without such knowledge (Influenza: *M*_with_ = 3.83 vs. *M*_without_ = 3.14, t(98) = 3.02, *p* = 0.003, Cohen’s *d* [95% CI] = 0.63 [0.21, 105]; Pneumococci: *M*_with_ = 3.75 vs. *M*_without_ = 3.20, t(94) = 2.32, *p* = 0.022, Cohen’s *d* [95% CI] = 0.49 [0.07, 0.91]). Perceived susceptibility did not differ between participants with and without knowledge of increased cardiovascular risk. Individuals who knew about the cardioprotective benefits of vaccination rated the usefulness of influenza vaccination (*M*_with_ = 4.15 vs. *M*_without_ = 3.60, t(98.8) = 2.38, *p* = 0.020, Cohen’s *d* [95% CI] = 0.46 [0.07, 0.85]) and pneumococcal vaccination (*M*_with_ = 4.18 vs. *M*_without_ = 3.66, t(95) = 2.26, *p* = 0.026, Cohen’s *d* [95% CI] = 0.47 [0.06, 0.88]) significantly higher. No differences were found regarding perceived vaccination risk in patients with vs. without knowledge on cardioprotective benefits. Further details can be found in Supplement Tables S1 and S2.

Regarding vaccination intention, a linear regression analysis (Table 2) adjusted for age, sex, and educational level showed that knowledge about the cardioprotective benefit of the vaccination was associated with a higher intention to receive influenza (*b*_y_ = 0.46, *p* = 0.024) and pneumococcal vaccination (*b*_y_ = 0.45, *p* = 0.034). In contrast, knowledge about the increased cardiovascular risk associated with acute respiratory infections was not significantly related to vaccination intention (Table 3), neither for influenza (*b*_y_ = 0.08, *p* = 0.699) nor for pneumococci (*b*_y_ = 0.34, *p* = 0.091). The proportions of variance explained by the models were *R*^2^ = 0.22 for influenza and *R*^2^ = 0.21 for pneumococcal vaccination. We conducted standard regression diagnostics for all models, and detailed results are provided in the Supplementary Materials.

## 4. Discussion

Our survey study indicates that two thirds of respondents were aware of the increased cardiovascular risk associated with acute respiratory infections, whereas substantially fewer recognized the cardioprotective benefits of the corresponding vaccinations. Individuals with pre-existing cardiovascular disease were more likely to be aware of the cardioprotective effects of vaccination, while no such difference was observed for knowledge about the infection-related increase in cardiovascular risk. Furthermore, knowledge of the elevated cardiovascular risk was associated with a higher perceived severity of influenza and pneumococcal infections, whereas knowledge of the cardioprotective benefits of vaccination was linked to a higher perceived usefulness of the vaccines. Notably, only knowledge about the cardioprotective benefits of vaccination—rather than knowledge about the cardiovascular risks posed by acute respiratory infections—was significantly associated with vaccination intention. These patterns were consistent for both influenza and pneumococcal vaccination.

Our study results differ markedly from previous research with respect to the prevalence of knowledge regarding the increased cardiovascular risk associated with acute respiratory infections and the cardioprotective benefits of vaccination. For example, in an Australian study involving patients with pre-existing cardiovascular disease, only 35.8% were aware of the elevated cardiovascular risk, and merely 27.4% recognized the cardioprotective effect of the seasonal influenza vaccination [27]. In another study among elderly patients with cardiovascular diseases, even none of the participants were aware of this association [28]. Possible reasons for the higher level of knowledge observed in our study might be attributable to the multimorbidity and the context of hospitalization in the geriatric patients included. We hypothesize that older adults with multiple chronic conditions have frequent interactions with healthcare professionals, which might increase exposure to counselling on infection-related cardiovascular risks and the benefits of vaccination, and that their current geriatric hospitalization may further heighten their perceived vulnerability and receptiveness to preventive information. For pneumococci, we did not find any studies addressing awareness of the infection-related increase in cardiovascular risk associated with pneumococcal disease or the cardioprotective benefit of pneumococcal vaccination. However, existing evidence shows that general awareness of pneumococcal vaccines is low among adults at high risk for pneumococcal disease, which appears to contribute to the persistently low uptake of pneumococcal immunization in this population [29].

Although knowledge prevalences were higher in our study population, a substantial information gap remains. This has important implications, as that lack of knowledge about the cardioprotective effects of influenza vaccination is linked to low-risk perception among patients with cardiovascular diseases [27,30]—a finding that is supported by our results. Conversely, providing information about the cardioprotective benefits of influenza vaccination has been shown to increase vaccination willingness and can enhance uptake in previous studies [21,28,31]. For example, a nationwide Danish intervention study demonstrated that a reminder letter explicitly emphasizing the cardioprotective effects of influenza vaccination significantly increased vaccination uptake [21]. Similarly, in another study, even a brief verbal education delivered directly by the physician had a substantial impact on vaccination behavior, leading 51% of previously unvaccinated patients to accept influenza immunization [28]. Together, the findings of these previous studies suggest that communicating the cardioprotective benefits of influenza vaccination represents an important strategy for improving influenza vaccine acceptance among populations at increased cardiovascular risk. Such efforts may have benefits extending beyond individual vaccination behavior, as infections impose a substantial clinical and economic burden on older adults and healthcare systems [32].

In contrast, comparable evidence for pneumococcal vaccination is currently lacking. We found no studies which have systematically evaluated if knowledge on cardioprotective benefits can increase risk perception and vaccination intention. This represents an important evidence gap, particularly given the substantially lower pneumococcal vaccination coverage observed in many countries despite longstanding recommendations for older adults and individuals at increased cardiovascular risk. Future research should therefore further explore the potential effect of gain-framed communication strategies to include the cardiovascular benefits of pneumococcal vaccination.

Beyond pneumococcal vaccination, our findings highlight the potential relevance of communicating broader health benefits of preventive interventions, but future research is needed that investigates how such information can be translated into effective communication approaches at the individual, organizational, and public health level. At the individual level, tailored information may increase awareness of additional health benefits through gain-framed messaging [33] and thereby support informed decision-making. In Germany, the Health Data Utilization Act (Gesundheitsdatennutzungsgesetz, GDNG) [34] enables statutory health insurance funds to use claims data to identify insured individuals for whom vaccinations are likely to be indicated and to provide targeted health information. This provides an opportunity to communicate potential additional benefits of vaccination in a way tailored specifically to relevant risk groups, potentially allowing for more targeted communication than broad population-based campaigns [35]. At the organizational level, healthcare providers could incorporate these benefits into structured vaccination counselling, for example in primary care practices and pharmacies [36] which offer COVID-19 and Influenza vaccinations in Germany, and office-based reminder systems which are currently implemented in around 40% of primary care practices in Germany [37]. At the public health level, evidence on broader health benefits could inform targeted national awareness campaigns, including those conducted by the Federal Institute for Public Health (BIÖG) [16], which already targets older adults and risk groups in its vaccination campaigns. Importantly, these approaches should consider age-specific needs rather than focusing exclusively on older adults as the main target population. Previous research has shown that gain-framing effects depend on recipient characteristics [33]. Thus, younger individuals with cardiovascular risk factors or other chronic conditions may require different information and counselling approaches, whereas older adults may benefit from messages tailored to their specific risk profiles and preventive care needs. Future research should therefore investigate how age- and risk-specific communication can be effectively implemented across individual, organizational, and public health settings.

## 5. Strengths and Limitations

This study has several strengths. To our knowledge, it is the first investigation to examine awareness of the infection-related increase in cardiovascular risk associated with pneumococcal disease and the potential cardioprotective benefits of pneumococcal vaccination. By focusing on older adults and individuals with cardiovascular pre-existing conditions, the study targets a population for whom vaccination is particularly relevant. The inclusion of both influenza and pneumococcal vaccination enables cross-pathogen comparisons of awareness patterns and behavioral correlates. Furthermore, conducting the survey among hospitalized geriatric patients increases clinical relevance, as these individuals represent a key target group for preventive vaccination strategies.

Several limitations should be considered when interpreting the findings. The limited sample size restricts parameter precision, statistical power and generalizability. For the same reason, we did not adjust for multiple testing in order to avoid a further decrease in statistical power, fully acknowledging that the family-wise error rate exceeds the nominal alpha level of 5%. Recruitment from hospitalized geriatric patients and self-help groups may have introduced a selection bias, potentially overrepresenting individuals with higher healthcare contact and multimorbidity. As a consequence, the observed knowledge prevalences and vaccination intentions are likely higher than in the broader population and should not be generalized without caution. The cross-sectional study design precludes causal inference regarding the relationships between knowledge, risk perception, and vaccination intention, as no temporal ordering between these variables can be established. For example, individuals who were already more favorable toward vaccination, had previously been vaccinated, or had more frequent healthcare interactions may have been more likely to acquire or retain information about cardioprotective vaccination benefits. In addition, our set of covariates was limited to age, sex, and educational level and is likely incomplete. Residual confounding is therefore probable, and the observed associations should not be interpreted as causal effects. In particular, previous vaccination behavior, comorbidity and healthcare exposure represent important potential sources of residual confounding that could not be fully accounted for in our models. Missing data were handled using listwise deletion; given the low proportion of missing values (max. 5.9%), substantial bias is unlikely, although complete-case analysis cannot fully exclude the possibility of non-random missingness. All data were self-reported, which may be subject to recall bias or social desirability bias [38], particularly concerning vaccination status [39]. Moreover, the use of single-item measures to assess vaccination knowledge only captured narrowly defined factual understanding and does not provide a comprehensive assessment of a broader knowledge construct. Furthermore, collapsing incorrect and “I don’t know” responses into a single category may obscure potential differences between incorrect beliefs and uncertainty; however, a more detailed categorization was not feasible given the limited sample size. Additionally, knowledge levels may have been overestimated, as hospitalized patients could have recently received counselling or health information related to infection risks and vaccination.

## 6. Conclusions

Our findings indicate that awareness of the cardioprotective benefits of influenza and pneumococcal vaccination was associated with higher vaccination intention, whereas awareness of the cardiovascular risks associated with acute respiratory infections was not.

These findings indicate a potential relevance of cardioprotective benefit information for preventive communication, but the cross-sectional design does not allow conclusions about causality or whether increasing knowledge would increase vaccination intention or actual vaccine uptake. For influenza vaccination, the observed association provides a rationale for further investigation alongside evidence from previous intervention studies showing that targeted communication can increase vaccine uptake. For pneumococcal vaccination, whether communicating cardioprotective benefits can influence vaccination intention or uptake remains to be established. Future research should therefore evaluate whether gain-framed communication about cardioprotective vaccination benefits can influence vaccination behavior in controlled intervention studies.

## Figures and Tables

**Table 1:** Descriptive statistics for demographic data, knowledge, vaccination intention and patient vaccination rates (*n* = 101).

Variables	Missing	Characteristic
Age [in years], Mean (SD)	0	71.9 (16.7)
Sex [female], *n* (%)	1	67 (67.0)
Cardiovascular diseases [yes], *n* (%)	2	61 (61.6)
Educational level, *n* (%)	0	
low		23 (22.8)
intermediate		27 (26.7)
high		51 (50.5)
Knowledge about … [yes], *n* (%)		
the increased cardiovascular risk of acute respiratory infections	0	65 (64.4)
the cardioprotective benefits of vaccination	0	41 (40.6)
Vaccination intention, Mean (SD)		
Influenza	1	3.7 (1.7)
Pneumococci	6	3.0 (1.6)
Vaccination rate, *n* (%)		
Influenza	1	63 (63.0)
Pneumococci	7	48 (51.1)

Note: SD = standard deviation. *n* = absolute frequency. % = percentage.

**Table 2:** Linear regressions of vaccination intention for influenza and pneumococcal disease (scale 1 = very low to 5 = very high) depending on knowledge about the cardioprotective vaccination benefits.

	Influenza (*n* = 100)			Pneumococci (*n* = 94)		
Predictors	*b*_y_	[95% CI]	*p*-Value	*b*_y_	[95% CI]	*p*-Value
Age (in 10 years)	0.20	[0.08, 0.33]	0.002 *	0.24	[0.11, 0.37]	<0.001 *
Sex (Reference: male)	−0.06	[−0.45, 0.33]	0.769	−0.22	[−0.62, 0.18]	0.277
Educational level (Reference: low)						
intermediate	0.01	[−0.60, 0.61]	0.983	0.60	[−0.01, 1.21]	0.052
high	−0.20	[−0.69, 0.29]	0.423	0.15	[−0.35, 0.66]	0.549
Knowledge about cardio-protective benefits of vaccination (Reference: none)	0.46	[0.06, 0.85]	0.024 *	0.45	[0.04, 0.86]	0.034 *

Note: CI = confidence interval. Educational level = categorized highest educational attainment. Reading example: the y-standardized regression coefficient *b*_y_ for the predictor “knowledge about the cardioprotective benefits of vaccination” represents the change in standard deviations of vaccination intention—controlling for all other predictors—when such knowledge is present. * = significant at significance level α = 0.05.

**Table 3:** Linear regressions of vaccination intention for influenza and pneumococcal disease (scale 1 = very low to 5 = very high) depending on knowledge about the increased cardiovascular risk of acute respiratory infections.

	Influenza (*n* = 100)			Pneumococci (*n* = 94)		
Predictors	*b*_y_	[95% CI]	*p*-Value	*b*_y_	[95% CI]	*p*-Value
Age (in 10 years)	0.22	[0.10, 0.35]	<0.001 *	0.26	[0.13, 0.39]	<0.001 *
Sex (Reference: male)	−0.08	[−0.48, 0.32]	0.685	−0.22	[−0.62, 0.18]	0.279
Educational level (Reference: low)						
intermediate	−0.15	[−0.76, 0.45]	0.612	0.46	[−0.14, 1.05]	0.133
high	−0.30	[−0.80, 0.20]	0.243	0.02	[−0.48, 0.53]	0.926
Knowledge about increased cardiovascular risk of acute respiratory infections (Reference: none)	0.08	[−0.32, 0.47]	0.699	0.34	[−0.06, 0.74]	0.091

Note: CI = confidence interval. Educational level = categorized highest educational attainment. Reading example: the y-standardized regression coefficient *b*_y_ for the predictor “knowledge about the about increased cardiovascular risk of acute respiratory infections” represents the change in standard deviations of vaccination intention—controlling for all other predictors—when such knowledge is present. * = significant at significance level α = 0.05.

## Data Availability

The datasets used and/or analyzed during the current study are available from the corresponding authors on reasonable request. Access to the anonymized data might be granted following review and permission by the ethics commission and data protection officer of the Jena University Hospital.

## Supplementary Materials

The following supporting information can be downloaded at: https://www.mdpi.com/article/10.3390/vaccines14100901/s1, Figure S1: Diagnostic plots assessing model assumptions for the linear regression of influenza vaccination intention.; Figure S2: Diagnostic plots assessing model assumptions for the linear regression of influenza vaccination intention.; Table S1: Descriptive statistics and group comparisons for infection-related and vaccination-related aspects of risk perception for influenza depending on knowledge.; Table S2: Descriptive statistics and group comparisons for infection-related and vaccination-related aspects of risk perception for pneumococci depending on knowledge.
