# Supplementary material for: Cardioprotective Vaccination Effects: Association Between Vaccination Knowledge, Risk Perception, and Vaccination Intention in Older Patients with Pre-Existing Conditions
Source: Vaccines (Basel). 2026 Oct 14;14(10):901. doi: 10.3390/vaccines14100901 (PMC13709624; doi:10.3390/vaccines14100901)
Supplement: Supplementary file 1 [file vaccines-14-00901-s001.zip › vaccines-4542184-supplementary.pdf]

Supplement

Inhalt

Questionnaire 2
Supplementary Tables 4
Results for the Regression Diagnostics 5
References 7

Questionnaire

Demographics

1. How old are you?

   ____ years □ no response

2. What is your gender?

   □ male □ female □ divers □ no response

3. What is your highest level of education?

   □ Without school-leaving qualification

   □ Lower secondary school certificate (former GDR: “POS”, 8 or 9 years of schooling)

   □ Intermediate secondary school certificate (former GDR: “POS”, 10 years of schooling)

   □ General or subject-specific higher education entrance qualification

   □ University or university of applied sciences degree

   □ No response / Not specified

4. Do you have any chronic diseases?

   □ yes □ no □ no response

5. Have you ever had, or do you currently have, any of the following heart or vascular diseases?
   - Heart attack (myocardial infarction)
   - Stroke (cerebrovascular insult)
   - High blood pressure (arterial hypertension)
   - Cardiac arrhythmias (e.g., atrial fibrillation)
   - Heart failure
   - Peripheral arterial disease (PAD)
   - Coronary artery disease (CAD)

   □ yes □ no □ no response

Vaccination knowledge

6. Please indicate whether the following statements are **true**, **false**, or **if you do not know**.
   a. Vaccinations against pathogens causing acute respiratory infections (such as influenza or pneumococci) also protect against cardiovascular diseases, such as heart attacks or strokes.
   b. Acute respiratory infections, such as influenza and pneumonia, increase the risk of cardiovascular diseases, including heart attacks and strokes.

Vaccination status

7. Did you receive an influenza vaccination during the last autumn/winter season (2024/25)?

   □ yes □ no □ no response

8. Have you received a pneumococcal vaccination at any time during the past six years (pneumococci are bacteria that can cause pneumonia)?

   □ yes □ no □ no response

Risk perception (Influenza/Pneumococci)

9. How dangerous do you think the following pathogens are for you?

   (1-very low, 2-low, 3-moderate, 4-high, 5-very high, I don’t know)

   a. Influenza virus

   b. Pneumococci (bacteria that can cause pneumonia, for example)

10. What do you think is the probability that you will contract the following pathogens if you are **not** vaccinated?

(1-very low, 2-low, 3-moderate, 4-high, 5-very high, I don't know)

   a. Influenza virus
   b. Pneumococci (bacteria that can cause pneumonia, for example)

11. How dangerous do you think **vaccination** against the following pathogens is for you?

    (1-very low, 2-low, 3-moderate, 4-high, 5-very high, I don't know)

    a. Influenza virus
    b. Pneumococci (bacteria that can cause pneumonia, for example)
    c. Corona virus (COVID-19

12. How high do you estimate **the usefulness of vaccination** against the following pathogens for you?

    (1-very low, 2-low, 3-moderate, 4-high, 5-very high, I don't know)

    a. Influenza virus
    b. Pneumococci (bacteria that can cause pneumonia, for example)
    c. Corona virus (COVID-19

Vaccination intention

13. How likely is it that you will receive an influenza vaccination during the upcoming autumn/winter season (2025/26)?

    (1-very low, 2-low, 3-moderate, 4-high, 5-very high, I don't know)

14. How likely is it that you will receive a pneumococcal vaccination within the next six months?

    (1-very low, 2-low, 3-moderate, 4-high, 5-very high, I don't know)

Supplementary Tables

Table S1. Descriptive statistics and group comparisons for infection-related and vaccination-related aspects of risk perception for influenza depending on knowledge.

Risk Perception	With Knowledge		Without Knowledge		Comparison				
	n	*M* (SD)	*n*	M (SD)	Diff (SE)	*t*-value	*df*	*p*-value	Cohen's *d* (95% CI)
**Infection-related**									
Severity of Infection	64	3.83 (0.98)	36	3.14 (1.27)	0.69 (0.24)	3.02	98	0.003*	0.63 (0.21, 1.05)
Susceptibility for Infection	64	3.39 (1.06)	36	3.36 (1.07)	0.03 (0.22)	0.13	98	0.895	0.03 (-0.38, 0.44)
**Vaccination-related**									
Benefits of Vaccination	41	4.15 (0.96)	60	3.60 (1.36)	0.55 (0.23)	2.37	98.8	0.020*	0.46 (0.07, 0.85)
Risks of Vaccination	39	1.95 (1.12)	58	2.31 (1.25)	-0.36 (0.24)	-1.46	95	0.148	-0.30 (-0.71, 0.11)

Note: *M* = Mean. SD = Standard deviation. Diff = Mean difference. SE = Standard error. *df* = Degrees of freedom. CI = Confidence interval. Aspects of risk perception were measured on a scale from 1 = very low to 5 = very high. Knowledge about the increased cardiovascular risk of acute respiratory infections or the cardio protective benefit of vaccinations served as the grouping variable for infection related and vaccination related aspects of risk perception, respectively. Group comparisons were conducted using t-tests or robust Welch t-tests in cases of heteroscedasticity. * = significant at significance level α = 0.05.

Table S2. Descriptive statistics and group comparisons for infection-related and vaccination-related aspects of risk perception for pneumococci depending on knowledge.

Risk Perception	With Knowledge		Without Knowledge		Comparison				
	n	*M* (SD)	*n*	*M* (SD)	Diff (SE)	*t*-value	*df*	*p*-value	Cohen's *d* (95% CI)
**Infection-related**									
Severity of Infection	61	3.75 (0.99)	35	3.20 (1.32)	0.55 (0.26)	2.32	94	0.022*	0.49 (0.07, 0.91)
Susceptibility for Infection	61	3.25 (1.11)	36	3.31 (1.14)	-0.06 (0.24)	-0.25	95	0.800	-0.05 (-0.47, 0.36)
**Vaccination-related**									
Benefits of Vaccination	39	4.18 (0.91)	58	3.66 (1.24)	0.52 (0.22)	2.26	95	0.026*	0.47 (0.06, 0.88)
Risks of Vaccination	36	2.08 (1.23)	55	2.29 (1.21)	-0.21 (0.26)	-0.79	89	0.429	-0.17 (-0.59, 0.25)

Note: *M* = Mean. SD = Standard deviation. Diff = Mean difference. SE = Standard error. *df* = Degrees of freedom. CI = Confidence interval. Aspects of risk perception were measured on a scale from 1 = very low to 5 = very high. Knowledge about the increased cardiovascular risk of acute respiratory infections or the cardio protective benefit of vaccinations served as the grouping variable for infection related and vaccination related aspects of risk perception, respectively. Group comparisons were conducted using t-tests or robust Welch t-tests in cases of heteroscedasticity. * = significant at significance level α = 0.05.

Results for the Regression Diagnostics

Regression Diagnostics for the Influenza Vaccination Intention Model

To assess model assumptions for the linear regression predicting influenza vaccination intention, we conducted standard diagnostic procedures including residual plots, tests for normality and homoscedasticity, evaluation of multicollinearity, and inspection of influential observations. The residuals-vs-fitted and scale-location plots indicated slight non-linearity and minor variance heterogeneity, but no systematic patterns suggesting substantial violations (Figure S1). The Shapiro–Wilk test showed deviations from normality ($W = 0.94$, $p < 0.001$), which is common in models using scaled Likert-type outcomes. The Breusch–Pagan test did not provide evidence of heteroscedasticity ($BP = 2.67$, $df = 5$, $p = 0.75$). Variance inflation factors were low (all VIF < 2.2), indicating no relevant multicollinearity. Cook's distance identified several observations above the conventional threshold (4/n), but none exerted undue influence on the model or materially affected the results. Taken together, we found evidence for non-normal distributions of residuals in the multiple linear regression, but no evidence for heteroscedasticity. As Schmidt and Finan (1) outline, linear regressions are generally robust to non-normality but sensitive to heteroscedasticity. Given that heteroscedasticity was not detected and deviations from normality were minor and expected for Likert-type outcomes, we consider the use of linear regression models in our study to be tenable and appropriate.

Figure S1. Diagnostic plots assessing model assumptions for the linear regression of influenza vaccination intention.

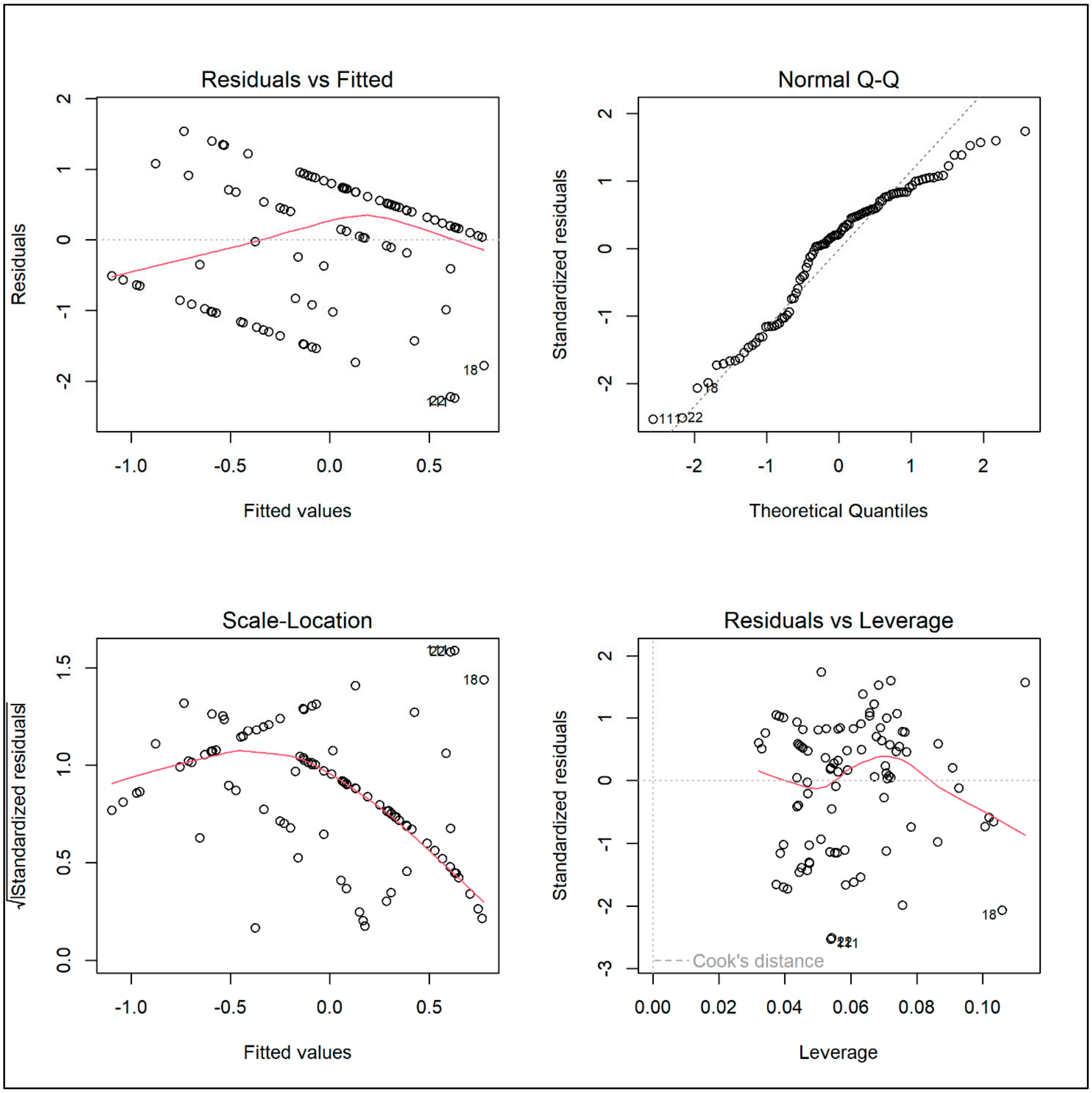

Regression Diagnostics for the Pneumococcal Vaccination Intention Model

To assess model assumptions for the linear regression predicting pneumococcal vaccination intention, we conducted standard diagnostic procedures including residual plots, tests for normality and homoscedasticity, evaluation of multicollinearity, and inspection of influential observations. The residuals-vs-fitted and scale-location plots indicated slight non-linearity and minor variance heterogeneity, but no systematic patterns suggesting major violations (Figure S2). The Shapiro–Wilk test showed deviations from normality (W = 0.96, p = 0.003), which is common in models using scaled Likert-type outcomes. The Breusch–Pagan test did not provide evidence of heteroscedasticity (BP = 8.98, df = 5, p = 0.11). Variance inflation factors were low (all VIF < 2.2), indicating no relevant multicollinearity. Cook's distance identified several observations above the conventional threshold (4/n), but none exerted undue influence on the model or materially affected the results. Taken together, we found evidence for non-normal distributions of residuals in the multiple linear regression, but no evidence for heteroscedasticity. As Schmidt and Finan (1) outline, linear regressions are generally robust to non-normality but sensitive to heteroscedasticity. Given that heteroscedasticity was not detected and deviations from normality were minor and expected for Likert-type outcomes, we consider the use of linear regression models in our study to be tenable and appropriate.

Figure S2. Diagnostic plots assessing model assumptions for the linear regression of influenza vaccination intention.

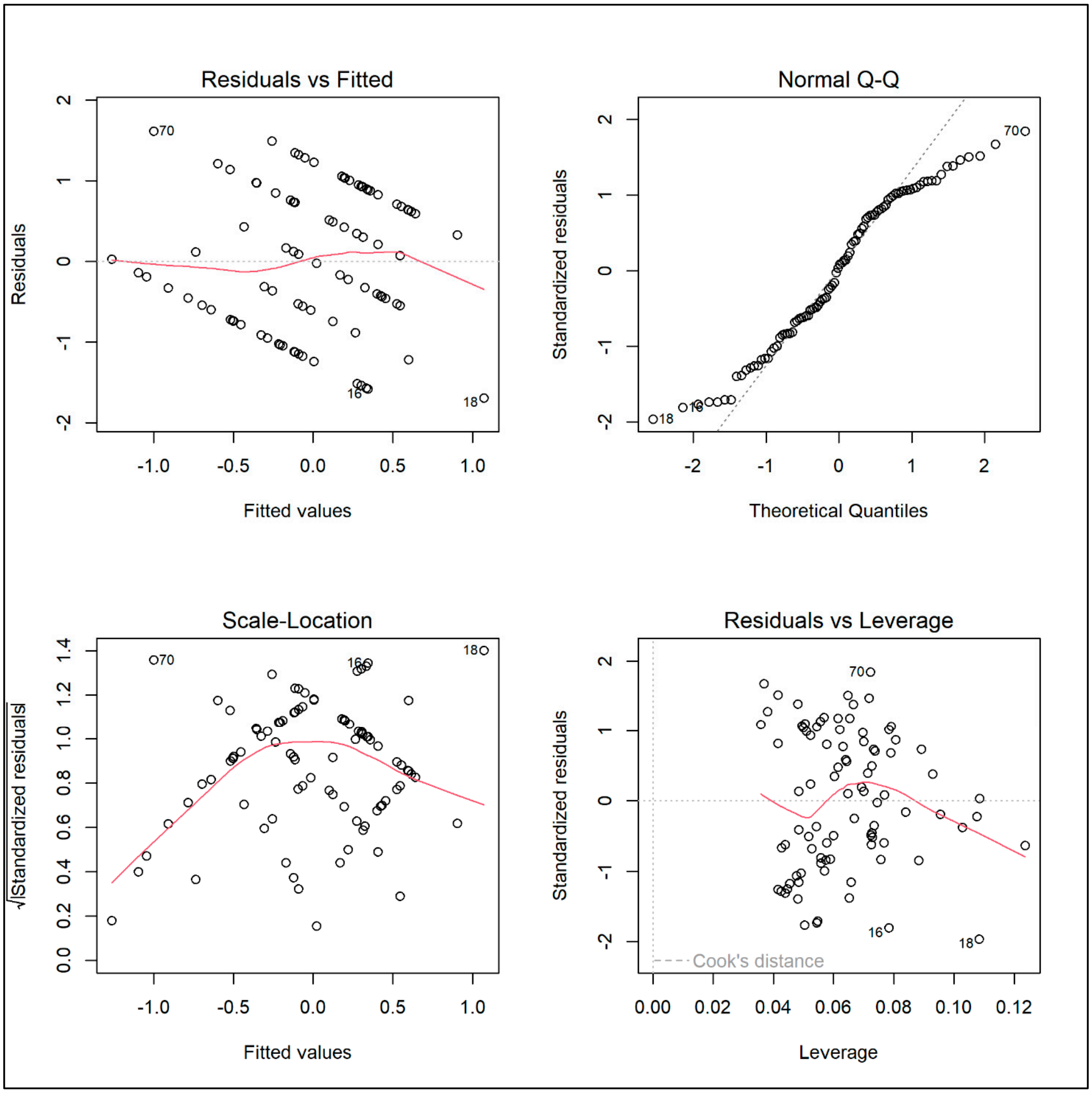

References

1. Schmidt AF, Finan C. Linear regression and the normality assumption. *J Clin Epidemiol.* **2018**, *98*, 146–151.
